# Burosumab in adults with X-linked hypophosphatemia: real-world experience from a retrospective study in Sydney

**DOI:** 10.1093/jbmrpl/ziae132

**Published:** 2025-12-06

**Authors:** Shejil Kumar, Christian M Girgis, Brian Tran, Malu Alvarez, Roderick J Clifton-Bligh

**Affiliations:** Department of Endocrinology, Royal North Shore Hospital, Sydney, NSW 2065, Australia; Faculty of Medicine & Health, University of Sydney, Sydney, NSW 2050, Australia; Department of Endocrinology, Westmead Hospital, Sydney, NSW 2145, Australia; Westmead Institute for Medical Research, Westmead Hospital, Sydney, NSW 2145, Australia; Faculty of Medicine & Health, University of Sydney, Sydney, NSW 2050, Australia; Department of Endocrinology, Westmead Hospital, Sydney, NSW 2145, Australia; Westmead Institute for Medical Research, Westmead Hospital, Sydney, NSW 2145, Australia; Faculty of Medicine & Health, University of Sydney, Sydney, NSW 2050, Australia; Department of Endocrinology, Westmead Hospital, Sydney, NSW 2145, Australia; Department of Endocrinology, Royal North Shore Hospital, Sydney, NSW 2065, Australia; Department of Endocrinology, Royal North Shore Hospital, Sydney, NSW 2065, Australia; Faculty of Medicine & Health, University of Sydney, Sydney, NSW 2050, Australia; Cancer Genetics Laboratory, Kolling Institute of Medical Research, Sydney, NSW 2065, Australia

**Keywords:** burosumab, hypophosphatemia, X-linked hypophosphatemia, X-linked hypophosphatemic rickets, Fgf-23

## Abstract

X-linked hypophosphatemia (XLH) is a chronic disabling hereditary musculoskeletal disorder associated with inactivating *PHEX* mutations and elevated circulating FGF-23 concentrations. In a placebo-controlled trial of adults with XLH, burosumab (anti-FGF-23 antibody) demonstrated durable improvements in phosphate concentrations, and self-reported stiffness and physical limitation. However, real-world data regarding burosumab efficacy and tolerability in adults with XLH are lacking. A retrospective audit was performed of patients (age ≥18-years) who commenced 4-weekly subcutaneous burosumab for XLH at Royal North Shore and Westmead Hospitals, Sydney, between January 2021 and June 2024. Patients were managed per standard clinical care and burosumab dose adjusted as necessary according to manufacturer instructions. Electronic medical records were reviewed to collate data regarding patient demographics, XLH-related complications and prior treatment, burosumab dosage and side effects, and pre- and post-burosumab biochemistry and Western Ontario and McMaster Universities Arthritis Index (WOMAC) scores. Of the 13 adults with XLH, all had hypophosphatemia before commencing burosumab (mean 0.64 ± 0.08 mmol/L). Mean WOMAC scores demonstrated baseline impairments in stiffness, pain, and physical limitation. Burosumab was administered for median 15 months during follow-up (median dose 70 mg). Hypophosphatemia resolved in all patients within 3 months of burosumab (mean 1.03 ± 0.38 mmol/L). Two patients developed hyperphosphatemia 2 weeks after commencing burosumab requiring dose reduction. One patient ceased burosumab in the setting of hypercalcemia and constipation secondary to pre-existing tertiary hyperparathyroidism. Adverse events were mild, including transient musculoskeletal discomfort (*n* = 4), restless legs (*n* = 2), injection site reaction (*n* = 2), and headache (*n* = 1). Repeat WOMAC within 12 months of commencing burosumab (*n* = 9) demonstrated clinically meaningful improvements in stiffness (-33.3 ± 12.5, *p*<.001) and physical function (−14.3 ± 16.2, *p*=.029). This study reports real-world outcomes of adults with XLH treated with burosumab. Clinical experience from 2 centers in Sydney supports trial findings that burosumab is well-tolerated and associated with improved serum phosphate concentrations and self-reported stiffness and physical function.

## Introduction

X-linked hypophosphatemia (XLH) is a chronic disabling hereditary musculoskeletal disorder associated with inactivating mutations in *PHEX*. XLH is the commonest genetic cause of hypophosphatemic rickets with an estimated incidence of ~1/20 000-70 000.^1^ XLH is characterized by excess circulating fibroblast growth factor-23 (FGF-23) concentrations and manifests with chronic hypophosphatemia, renal phosphate wasting, and multiple skeletal complications, including short stature, bone deformities (such as leg bowing), which often require corrective surgery, musculoskeletal pain and stiffness, early-onset osteoarthritis, enthesopathy, muscle weakness and fatigue, dental complications, fractures, and impaired quality of life.^2-5^ Conventional treatment for XLH includes multi-daily dosing of oral phosphate and active vitamin D, often limited by intolerance (eg, gastrointestinal), treatment-related complications (eg, hyperparathyroidism, nephrolithiasis), and poor adherence.^3^

Burosumab (anti-FGF-23 monoclonal antibody) targets the primary pathophysiology of XLH. A phase III randomized placebo-controlled trial of 4-weekly subcutaneous burosumab in symptomatic adults with XLH (*n* = 134) demonstrated durable improvements in phosphate concentrations, and self-reported joint stiffness and physical function with up to 3 years of treatment.^6-9^ Most burosumab-treated patients achieved normalization of phosphate levels (94.1% vs 7.6%, *p*<.001). Burosumab was associated with significant improvements in self-reported stiffness and physical function (using the Western Ontario and McMaster Universities Arthritis Index [WOMAC] scale) and faster gait speed (measured by 6-min walk distance). Burosumab was well-tolerated and reported side effects were balanced between active treatment and placebo controls, other than for injection site reactions (~12%). Serious adverse events, treatment interruption, and discontinuation were rare.

There have been few real-world data regarding burosumab efficacy and tolerability in adults with XLH published to date.^10^^,^^11^ In Australia, burosumab was made available via government subsidization for adults with XLH in October 2022 and hence real-world experience is limited. In this retrospective study we analyzed the effects of burosumab treatment on serum phosphate concentrations, patient-reported measures of pain, stiffness and physical function, and treatment-related side effects in adults with XLH managed in 2 outpatient specialist centers in Sydney.

## Materials and methods

A retrospective observational study was performed of consecutive patients (aged ≥18 years) transitioning from either nil or conventional treatment to 4-weekly subcutaneous burosumab injections for XLH at Royal North Shore and Westmead Hospitals, Sydney, between January 2021 and June 2024. Patients were eligible if they received at least 1 dose of burosumab with at least 1 result available for pre- and post-burosumab phosphate concentrations. Patients were excluded if unwilling or unable to provide informed consent for data collection. Diagnosis of XLH was made on the basis of serum phosphate concentrations chronically below the assay lower reference limit, evidence of renal phosphate wasting, and historical or recent radiographic findings consistent with rickets (eg, bowing of the legs). Patients were also required to meet at least 1 of the following criteria: presence of a pathogenic *PHEX* germline mutation, positive family history of X-linked inheritance, or plasma FGF-23 concentration above the mid-point of the assay reference range.

Burosumab was initiated at 1 mg/kg/dose (rounded to nearest 10 mg, up to maximal dose of 90 mg) with treatment interruptions or dose adjustments as necessary according to manufacturer instructions (Crysvita®, Kyowa Kirin Ltd, Tokyo, Japan). Burosumab was administered by a healthcare professional (either a senior nurse affiliated with the Endocrinology clinic or another healthcare professional such as the patient’s general practitioner). Patients were managed according to routine clinical care including serial clinical assessment, monitoring of serum phosphate and other bone-related biochemistry, patient-reported measures of pain, stiffness and physical function. and assessment of gait speed.^12^^,^^13^ Timing and frequency of collection of these endpoints were not protocolized and were up to clinician discretion.

Two investigators independently collected data by performing electronic medical record review and any incongruence was resolved by unanimous decision. Data collated included patient demographics (age, sex), age at diagnosis of rickets/osteomalacia, family history of XLH (yes/no), *PHEX* sequencing results if performed (pathogenic/likely pathogenic vs other), history of XLH-related complications (yes/no for long limb bowing, corrective orthopedic limb surgery, musculoskeletal discomfort, osteoarthritis, enthesopathy, dental complications, secondary/tertiary hyperparathyroidism, fragility fractures), prior conventional treatment (yes/no), prior poor adherence/intolerance to conventional treatment (yes/no) and burosumab dose (mg 4-weekly), and duration of treatment (months) up until study completion. Prior to commencing burosumab, height (cm) was measured using stadiometer rounded to nearest 0.1 cm and weight (kg) using standard measuring scales rounded to nearest 0.1 kg. Body mass index (BMI) was calculated using the standard formula: BMI = weight (kg) / (height (m).^2^ Results were also collected for pre- and post-burosumab serum phosphate concentrations and various bone-related biochemical markers where available, including serum albumin-corrected calcium, alkaline phosphatase (ALP), intact parathyroid hormone (iPTH), and 25-hydroxyvitamin D_3_. Plasma intact FGF-23 concentrations (measured using the Diasorin Liaison XL assay)^14^ prior to burosumab commencement were also recorded. Renal phosphate wasting pre- and post-burosumab was assessed using tubular maximal reabsorption of phosphate to glomerular filtration rate (TmP/GFR). TmP/GFR was calculated using an online calculator developed by the Australian & New Zealand Bone & Mineral Society imputing plasma and urine phosphate and creatinine concentrations from a paired fasting blood sample and second void urine sample.^15^^,^^16^

Patient-reported stiffness, pain, and physical function limitation were assessed before and after burosumab treatment using the WOMAC questionnaire, which was originally designed for use in patients with osteoarthritis but has been validated for use in adults with XLH as a relevant clinical endpoint.^17^^,^^18^ WOMAC is a 24-item questionnaire assessing patients’ self-perceived degree of musculoskeletal pain (*n* = 5 items), stiffness (*n* = 2 items), and physical limitation (*n* = 17 items) experienced over the past 48 h. Responses are presented on a 5-point Likert scale (none = 0, mild = 1, moderate = 2, severe = 3, extreme = 4). The pain (/20), stiffness (/8), and physical limitation (/68) subscale scores and total score (/96) can be expressed as raw scores or each converted to a 100-point scale, with the higher scores indicating worse self-perceived health status. For comparison of WOMAC scores pre- and post-burosumab, absolute changes in subscales and total score were expressed using the 100-point scale. Age- and sex-specific normative ranges have been published in a large dataset of healthy volunteers and minimal clinically meaningful changes with treatment in XLH patients have been described.^19^^,^^20^ Gait speed was measured by distance (meters) ambulated in a continuous 6-min period. Frequency of burosumab-related side effects, dose reduction, treatment interruption, and discontinuation were recorded. Side effects of special interest included musculoskeletal discomfort (eg, pain, stiffness), restless legs, headache, nasopharyngitis, injection site reactions, and elevated serum phosphate concentration above the assay upper reference limit.

**Table 1:** Baseline patient characteristics and patient-reported outcomes.

**Characteristic/outcome**	**Total cohort (*n* = 13)**
**Age, years (at burosumab commencement)**	
**Median (IQR)**	34 (29-58)
**Range**	19-77
**Female, n (%)**	9 (69%)
**Age at diagnosis of rickets/osteomalacia (*n* = 10)**	
**At birth**	4 (40%)
**<5 years**	3 (30%)
**5–18 years**	1 (10%)
**>18 years**	2 (20%)
**Long limb bowing, n (%)**	11 (85%)
**Corrective orthopedic limb surgery, n (%)**	9 (69%)
**Musculoskeletal discomfort, n (%)**	11 (85%)
**Osteoarthritis, n (%)**	9 (69%)
**Enthesopathy, n (%)**	7 (54%)
**Dental complications, n (%)**	12 (92%)
**Secondary/tertiary hyperparathyroidism, n (%)**	3 (23%)
**Prior fractures, n (%)**	6 (46%)
**Positive family history, n (%)**	9 (67%)
**Confirmatory *PHEX* genetic test, n (%) (*n* = 5)**	4 (80%)
**Poor adherence/intolerance to conventional therapy, n (%)**	7 (54%)
**Height (cm), mean ± SD (*n* = 7)**	149 ± 7
**Weight (kg), mean ± SD (*n* = 8)**	62 ± 9
**BMI (kg/m^2^), mean ± SD (*n* = 8)**	30.3 ± 5.8
**Serum phosphate (mmol/L), mean ± SD**	0.64 ± 0.08
**Serum corrected calcium (mmol/L), mean ± SD**	2.28 ± 0.10
**Serum ALP (U/L), mean ± SD**	106 ± 43
**Urine TmP/GFR (mmol/L), mean ± SD (*n* = 10)**	0.51 ± 0.06
**Plasma FGF-23 (ng/L), median (IQR)**	121 (91)
**WOMAC, mean ± SD (*n* = 11) [raw scores]**	
**Pain (/20)**	8.6 ± 3.6
**Stiffness (/8)**	5.1 ± 1.3
**Physical functioning (/68)**	28.2 ± 15.2
**Total score (/96)**	41.0 ± 19.0
**WOMAC, mean ± SD (*n* = 11) [100-point scores]**	
**Pain (/100)**	43.2 ± 18.7
**Stiffness (/100)**	63.9 ± 15.9
**Physical functioning (/100)**	41.5 ± 22.3
**Total score (/100)**	42.7 ± 19.8
**6-min walk test (meters), mean ± SD (*n* = 5)**	269 ± 142

IQR = interquartile range; BMI = body mass index; SD = standard deviation; *PHEX* = phosphate-regulating endopeptidase homolog X-linked; ALP = alkaline phosphatase; TmP/GFR = tubular maximal reabsorption of phosphate to glomerular filtration rate; FGF-23 = fibroblast growth factor-23; WOMAC = Western Ontario and McMaster Universities Arthritis Index. Normal-range for biochemical outcomes as follows: Serum phosphate (0.75-1.50 mmol/L); serum corrected calcium (2.10-2.60 mmol/L); serum ALP (30-110 U/L); serum FGF-23 (23.2-95.4 ng/L); urine TmP/GFR in adults aged >18 years (0.70-1.35 mmol/L). Confirmatory *PHEX* genetic test result refers to finding of pathogenic or likely pathogenic variant in *PHEX* gene on germline sequencing.

WOMAC raw and 100-point subscale and total scores were calculated based on prior literature,^9^ with the higher scores indicating worse self-reported impairment.

The study was performed in accordance with the Declaration of Helsinki. The protocol and participant consent form were approved by the Northern Sydney Local Health District (NSLHD) Human Research Ethics Committee (HREC) (Ref: 2024/ETH00293). All participants signed informed consent prior to data collection. Consent was obtained during a routine outpatient follow-up appointment by a study investigator not directly involved in the participant’s management.

## Statistical analysis

Statistical analyses were performed using SPSS statistical software (version 28.0). Baseline characteristics were tabulated and presented as either frequency or distribution using mean (±SD) or median (IQR) for normative and non-normative data, respectively (normality assessed using Shapiro Wilk test). Changes in outcome means after burosumab treatment were assessed using paired samples *t*-test. The pre-burosumab result was taken as the latest recorded result prior to commencing burosumab, and post-burosumab result as the earliest recorded result after commencing burosumab. A 2-sided *p*-value of <0.05 was considered statically significant.

**Table 2:** Burosumab dosage characteristics, treatment duration, and side effects.

Case number	Age at initiation (years)	Initial dose (mg)	Final dose (mg)	Treatment duration (months)	Side effects
**1**	19	70	70	10	Injection site reaction, headache
**2**	41	70	70	15	Transient musculoskeletal pain, restless legs
**3**	29	80	80	15	Transient musculoskeletal pain
**4**	61	70	70	18	Nil
**5**	34	70	70	12	Nil
**6**	34	90	90	18	Transient musculoskeletal pain
**7**	28	50	10	16	Restless legs
**8**	37	60	60	9	Injection site reaction
**9**	54	50	50	6	Nil
**10**	29	70	40	36	Transient musculoskeletal pain, hyperphosphatemia
**11**	64	90	Ceased	6	Hypercalcemia, constipation
**12**	77	90	Ceased	4	Hyperphosphatemia
**13**	30	70	70	39	Leg cramps

## Results

Thirteen adults with XLH were commenced on burosumab during the study period (range 19-77 years, 69% female). Most had prior intolerance and/or poor adherence with conventional treatments. Musculoskeletal complications were frequent including long limb bowing needing corrective surgery, musculoskeletal pain, osteoarthritis, enthesopathy, fractures, and dental complications (Table 1). *PHEX* genetic testing was performed in 5 patients, of whom 4 had a pathogenic/likely pathogenic *PHEX* variant and 1 had no *PHEX* or other variant (eg, FGF-23) identified. Many who had BMI recorded were overweight (25%) or obese (50%). All patients had hypophosphatemia prior to commencing burosumab (mean 0.64 ± 0.08 mmol/L, normal-range 0.75-1.50 mmol/L). Five patients (38%) had elevated serum ALP concentrations pre-burosumab. All patients with available TmP/GFR results demonstrated renal phosphate wasting. All patients had elevated or inappropriately normal FGF-23 concentrations at baseline. Mean WOMAC scores demonstrated baseline impairments in stiffness, pain, and physical function^19^ (Table 1).

**Figure 1:**
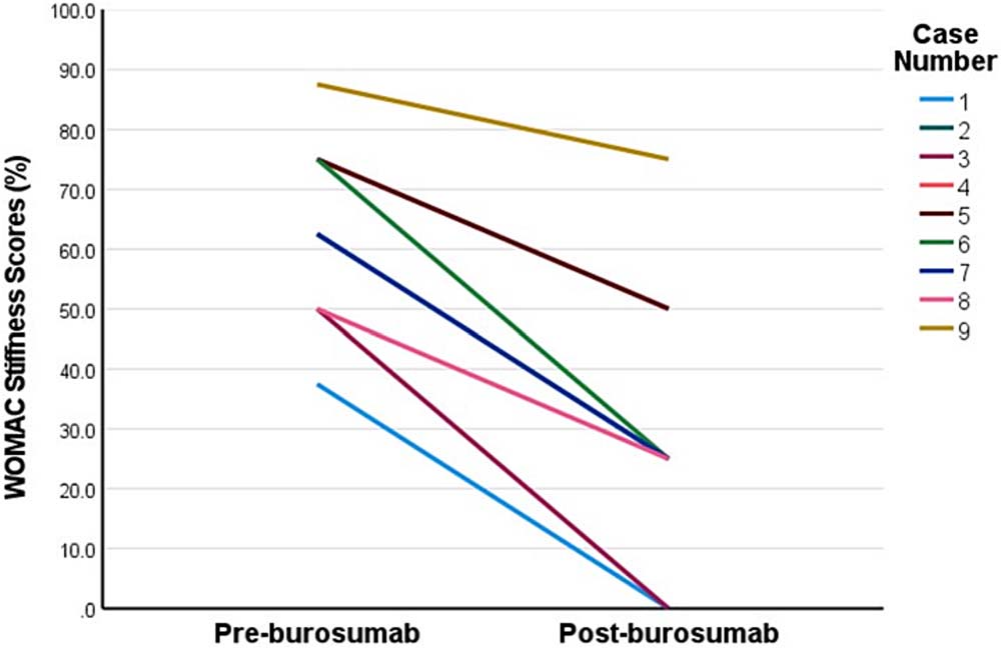
Individual patient responses in Western Ontario and McMaster Universities Arthritis Index (WOMAC) stiffness subscale scores pre- and post-burosumab. This line graph plots individual patient responses in WOMAC stiffness subscale scores (on 100-point scale) from pre-burosumab to post-burosumab (median 9 months). The higher scores indicate worse self-perceived stiffness. Two pairs of cases had the same response scores (cases 2 and 7 and cases 4 and 5). Minimum clinically meaningful change is defined as ≥10-15-point decrease for stiffness subscale.^20^

**Figure 2:**
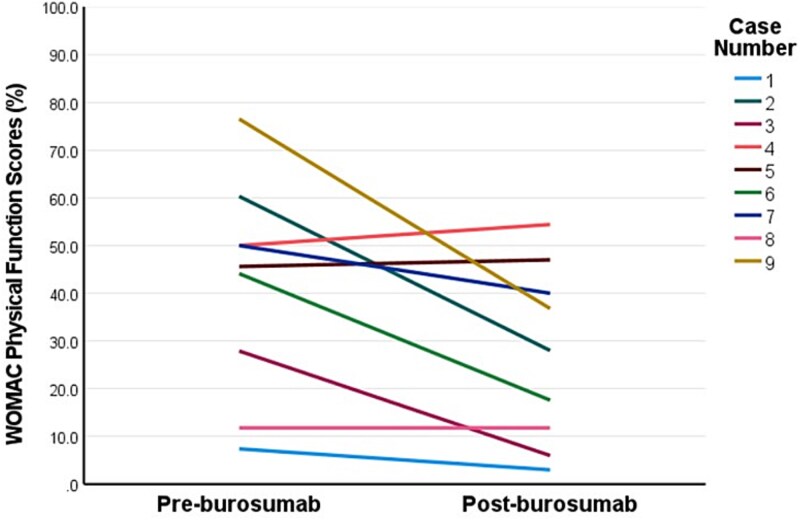
Individual patient responses in Western Ontario and McMaster Universities Arthritis Index (WOMAC) function subscale scores pre- and post-burosumab. This line graph plots individual patient responses in WOMAC function subscale scores (on 100-point scale) from pre-burosumab to post-burosumab (median 9 months). The higher scores indicate worse self-perceived functional limitation. Minimum clinically meaningful change is defined as ≥8-10-point decrease for function subscale.^20^

**Figure 3:**
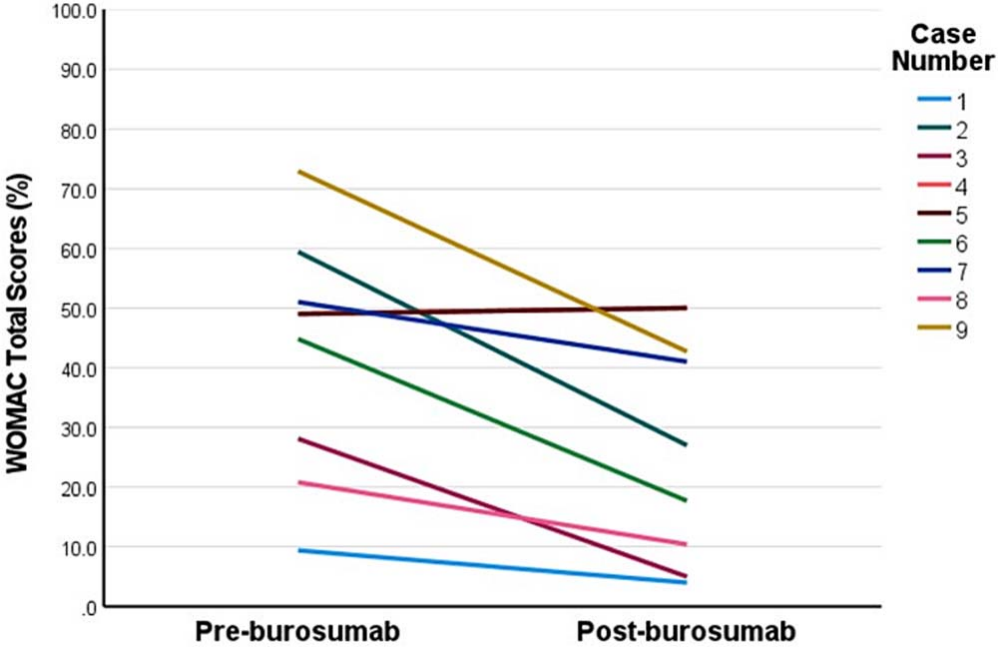
Individual patient responses in Western Ontario and McMaster Universities Arthritis Index (WOMAC) total scores pre- and post-burosumab. This line graph plots individual patient responses in WOMAC total scores (on 100-point scale) from pre-burosumab to post-burosumab (median 9 months). The higher scores indicate worse self-perceived health status. One pair of cases had the same response scores (cases 4 and 5). Minimum clinically meaningful change is defined as ≥10-point decrease for total score.^20^

**Figure 4:**
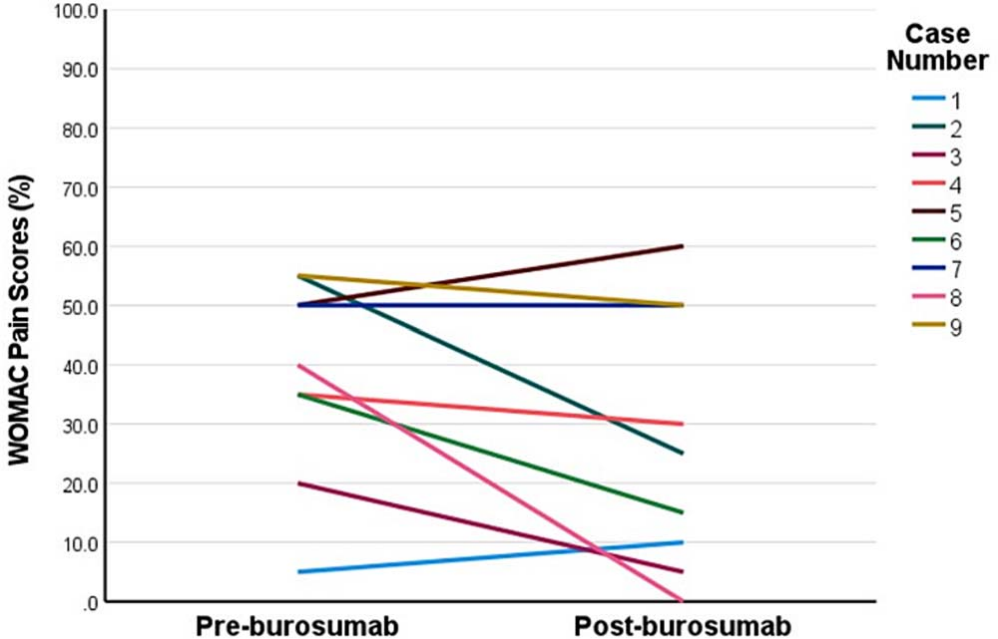
Individual patient responses in Western Ontario and McMaster Universities Arthritis Index (WOMAC) pain subscale scores pre- and post-burosumab. This line graph plots individual patient responses in WOMAC pain subscale scores (on 100-point scale) from pre-burosumab to post-burosumab (median 9 months). The higher scores indicate worse self-perceived pain. Minimum clinically meaningful change is defined as ≥11-point decrease for pain subscale.^20^

Two patients commenced burosumab via compassionate access prior to approval of government-funded access in Australia, after which a further 11 patients were commenced on burosumab. The median burosumab dose at initiation was 70 mg every 4 weeks, and median duration of burosumab was 15 months (range 4-39) (Table 2). All patients experienced resolution of hypophosphatemia within 3 months of commencing burosumab (mean 1.03 ± 0.38 mmol/L). Two patients developed elevated serum phosphate concentrations 2 weeks after commencing burosumab (1.63 mmol/L, 2.03 mmol/L), 1 of whom required brief interruption and dose reduction. Restless legs in 1 patient improved with 6-month dose interruption after which burosumab was recommenced at a reduced dose. One patient ceased burosumab in the setting of hypercalcemia and constipation secondary to pre-existing tertiary hyperparathyroidism, with pre- and post-burosumab corrected calcium (2.52 mmol/L; 2.67 mmol/L) and PTH concentrations, respectively (10.1 pmol/L; 13.7 pmol/L, normal-range: 1.6-7.2 pmol/L). One patient ceased burosumab in setting of poor adherence. Three patients had recurrence of hypophosphatemia at the end of the dosing interval. Side effects included transient musculoskeletal discomfort (*n* = 4), restless legs (*n* = 2), injection site reaction (*n* = 2), and headache (*n* = 1) of which none were severe. Repeat WOMAC assessment (*n* = 9) was conducted at median 9 months after commencing burosumab (range 3-12 months). Patients demonstrated significant clinically meaningful improvements in stiffness subscale (−33.3 ± 12.5 points, *p*<.001), physical function subscale (−14.3 ± 16.2 points, *p*=.029), and total score (−15.2 ± 13.2 points, *p*=.009) (Figures 1**-**3), while changes in pain subscale were not significant (−11.1 ± 16.5 points, *p*=.079) (Figure 4). Changes in 6-min walk distance were not analyzed due to insufficient baseline and follow-up data.

## Discussion

This retrospective cohort study provides real-world data of adults with XLH treated with burosumab, and the first from the Asia-Pacific region. We assessed a typical population of adults with XLH with impairments in self-perceived stiffness, pain, and physical limitation despite prior exposure to conventional treatments. Burosumab treatment was associated with early resolution of hypophosphatemia in all cases. Self-perceived stiffness improved considerably within the first 12 months of treatment. Overall findings were consistent with the pivotal placebo-controlled phase III randomized clinical trial (RCT) indicating burosumab is safe and effective in adults with XLH in the real-world setting.^6^

Our cohort consisted of 13 adults with numerous complications associated with XLH and/or prior conventional treatment. Musculoskeletal discomfort (such as pain and stiffness), osteoarthritis, and enthesopathy were frequent; this is consistent with several large cohorts of adults with XLH in which musculoskeletal features become more prevalent with age, predominantly affect load-bearing lower limb joints (such as the knee and hip), and contribute considerably to overall disease burden.^1^^,^^4^^,^^6^^,^^21-23^ Baseline characteristics of our cohort were similar to the RCT cohort in age, sex distribution, serum phosphate concentrations, TmP/GFR, and WOMAC scores, although our cohort did appear to have less marked self-perceived pain (43.2 points vs 49.3 points).^8^ For inclusion in the RCT, participants required the presence of skeletal pain (score of ≥4 on brief pain inventory), which may explain this difference from our cohort.^6^ Although our patients were not specifically required to be symptomatic of XLH to be commenced on burosumab and included in our study, all our patients displayed pre-treatment impairments in self-perceived health and functional status. Almost all patients had pain, stiffness, and functional limitation subscale scores above the age- and sex-specific 99th percentile range (12/13, 12/13, and 13/13, respectively), in comparison with established normative data.^19^

Less than half of our cohort had recorded *PHEX* genetic testing results. Evidence of *PHEX* genetic testing was only recorded if available in clinical documentation or results were scanned into the electronic medical record, and it is possible some *PHEX* testing was not captured. Further, a majority (11/13) accessed burosumab via the pharmaceutical benefits scheme for which identification of *PHEX* variant is not necessary if patients satisfy other clinical, biochemical, and radiographic criteria for a diagnosis of XLH. All patients met these diagnostic criteria exhibiting low serum phosphate, inappropriately elevated FGF-23 concentration above the mid-reference range, and evidence of renal phosphate wasting prior to commencing burosumab. Patients had mean BMI 30 kg/m^2^ with 75% having an elevated BMI above 25 kg/m^2^. Frequently elevated BMI has been reported in other adult XLH cohorts with potential contributors including short stature and sedentary lifestyle secondary to functional limitations.^1^^,^^6^^,^^21^^,^^24^ Waist circumference and other anthropometric measures (eg, body composition using dual-energy X-ray absorptiometry) were not performed during standard clinical care in our patients.

After commencing burosumab, all patients had resolution of hypophosphatemia at the earliest serum phosphate measurement performed, usually within the first month, and in all cases within the first 3 months. This is consistent with data from the phase III RCT in which 94.1% of burosumab-treated patients achieved a mean serum phosphate concentration above the lower reference limit when taken at mid-dose timepoints over the first 24-weeks of treatment (ie, 2 weeks post-dose when peak serum phosphate is expected).^6^ One patient in our cohort had elevated serum phosphate at the midpoint between the first and second dose (1.63 mmol/L) requiring dose interruption for 2 months and 50% dose reduction (from 70 mg to 40 mg) with all subsequent phosphate concentrations in the normal range. Hyperphosphatemia occurred in 6% of patients in the burosumab RCT and no cases occurred in a Brazilian real-world study (*n* = 19).^6^^,^^11^ Other biochemical markers of efficacy were inconsistently recorded post-burosumab commencement and hence were not included in our analyses, including treatment-related changes in TmP/GFR. Three patients experienced recurrence of hypophosphatemia during follow-up (0.44 mmol/L at 4 months, 0.71 mmol/L at 8 months, and 0.56 mmol/L at 11 months) when measured at the end of a dosing interval. In the phase III RCT, burosumab efficacy in maintaining normophosphatemia was less robust when measured at the end-of-dose interval, with one-third of patients having mean trough phosphate concentrations below the lower reference limit during the first 24 weeks.^6^ A slightly lower proportion maintained normal mean phosphate during extended follow-up to 96 weeks, with mean pre-dose concentrations being at or just below the lower reference limit during the second year of treatment.^7-9^ Low trough phosphate concentrations have been found in other real-world studies of burosumab in adults with XLH. An Italian study (*n* = 8) showed resolution of hypophosphatemia within the first 3 months of burosumab treatment^10^; although most maintained mid-dose normophosphatemia between 4 and 6 months of treatment, a majority (7/8) developed low trough phosphate concentrations. In another real-world study (*n* = 19) with mean 16 ± 8 months follow-up, there was significant increase in serum phosphate from 0.61 ± 0.14 to 0.86 ± 0.17 mmol/L from baseline to end of follow-up, although timing of phosphate measurement in relation to burosumab dosage was not specified.^11^ Hence, occurrence of low serum trough phosphate concentrations is not uncommon during burosumab treatment, particularly beyond the first 3 months of treatment. In cases of persistently low mid-dose phosphate concentrations, other factors should be considered such as poor adherence, inappropriate dosage, or concurrent tertiary hyperparathyroidism.

Patients on average demonstrated a ~20-40-point reduction in the WOMAC stiffness subscale score at a median 9 months after commencing burosumab (Figure 1). These improvements in self-perceived stiffness were well above the threshold to be considered clinically meaningful (>10-15-points).^20^ Albeit less marked, patients also had clinically meaningful improvements in self-perceived physical limitation (~15 points) (above the threshold of 8-10 points).^20^ Average improvements in pain scores (~11 points) were not statistically significant and were borderline for a clinically meaningful response. These responses in WOMAC self-perceived pain scores are similar to those seen over 48-weeks in the RCT, which only exceeded the threshold for clinically relevant change when treatment extended to 96-weeks, suggesting perhaps a longer treatment period is required to demonstrate this effect.^7^^,^^8^ Further, our cohort experienced less severe self-perceived pain at baseline than seen in the RCT cohort, which may further explain lack of improvement in in our cohort.^8^ Our finding of improved self-perceived stiffness, however, is consistent with other studies. In the phase III RCT, burosumab-treated patients had a mean 16-point improvement in WOMAC stiffness scores during a similar time-frame of 48 weeks, which plateaued during the second year of treatment.^8^^,^^9^ Our cohort was similar regarding age, sex distribution, frequency of musculoskeletal complications, and baseline mean WOMAC stiffness scores. A phase I/II open-label dose-escalation study of a similar cohort of adults with XLH demonstrated similar mean improvement of 35 points in stiffness subscale after 4 months of burosumab.^17^ The mechanisms of musculoskeletal stiffness in XLH are not well understood. Potential contributors include progressive enthesopathy (calcification of the entheses, ie, regions where ligaments and tendons attach to bone), accelerated osteoarthritis secondary to pre-existing long-term bone and joint deformities and misalignment, or muscle weakness.^25^ Enthesopathy is reported in majority of adults with XLH and prevalence increases with age in studies where enthesopathy was systematically assessed for using radiographic skeletal survey or multiple targeted X-ray scans.^6^^,^^26^^,^^27^ Preclinical studies using an XLH mouse model (*Hyp*) suggest enthesopathy may develop due to hyperplasia of fibrocartilaginous chrondrocytes as a maladaptive response to insertion of entheses into undermineralized bone in the presence of skeletal loading.^28-29^ Impaired 1,25-dihydroxyvitamin D signaling may also be implicated.^30^ The predilection of enthesopathy for load-bearing joints such as the knee, hip, and ankles supports this hypothesis.^1^^,^^4^^,^^6^^,^^21-23^ Conventional XLH treatment has not yet been shown to prevent enthesopathy in retrospective studies,^31^^,^^32^ and no studies to date have assessed burosumab treatment effects on enthesopathy. Our findings support the notion that burosumab leads to improvements in self-perceived stiffness in adults with XLH receiving burosumab in a real-world setting. Further research is warranted into the mechanisms for this treatment effect, particularly whether there is any regression of radiographic features of enthesopathy in load-bearing lower limb joints and whether this correlates with improvements in stiffness. The contribution of muscle dysfunction to stiffness and functional limitation in adults with XLH is also unclear. A recent prospective study (*n* = 10) of adults with XLH found no improvement in handgrip or lower limb strength or rates of ATP synthesis in the calf muscle using magnetic resonance spectroscopy after 3-months of burosumab treatment.^33^ This study had various limitations including small sample size and brief follow-up period. Further assessment of muscle effects of burosumab, and influence on improvements in stiffness and physical function are warranted.

Burosumab was well-tolerated in adults with XLH in the phase III RCT with low frequency of mild to moderately severe adverse events and no occurrence of treatment-related serious adverse events or dose-limiting toxicity. Our findings are consistent with this, with patients experiencing only mild to moderate side effects such as transient musculoskeletal discomfort, nasopharyngitis, restless legs, and injection site reactions. Dose reduction was required in 1 patient due to elevated serum phosphate concentration after which phosphate normalized; there was dose interruption and dose reduction in another patient due to restless legs; and another patient ceased burosumab due to constipation and hypercalcemia from pre-existing tertiary hyperparathyroidism. Given our study was retrospective, formal assessment of severity of side effects was not performed, although no side effects resulted in hospitalization and so it is unlikely any side effects were severe. In other real-world studies in adults with XLH, 1 study reported no serious side effects or treatment discontinuation with burosumab, while another study did not report on tolerability.^10^^,^^11^

Our study has several limitations. This was a retrospective analysis of real-world clinical experience managing adults with XLH using burosumab. Given frequency of assessing clinical and safety endpoints was at the discretion of the treating clinician, completeness of data for various endpoints was dependent on reporting in clinical documentation and availability of results. For this reason, there was also heterogeneity in timing of outcome assessments. Despite this, pre- and post-burosumab phosphate concentrations were available for all patients and pre- and post-WOMAC scores in the majority. Baseline WOMAC assessments in 2 patients were conducted retrospectively (with time lag of 1 month in 1 patient and 3 months in the other), which may have affected accuracy of these assessments. However, even after excluding these 2 cases, mean improvements in stiffness subscale (−30.4 ± 12.2, *p*<.001) and total score (−11.6 ± 12.6, *p*=.050) remained statistically significant and clinically meaningful. In the absence of a placebo-control cohort, we cannot exclude some component of placebo effect for improvements in patient-reported outcomes. Our cohort is small (*n* = 13 for phosphate outcomes, *n* = 9 for WOMAC outcomes), but still demonstrated statistically significant improvements in self-reported stiffness and functional limitation. Nonetheless, this is a relatively large real-world cohort of adults with XLH receiving burosumab and we have demonstrated improvements with treatment in clinically relevant endpoints including serum phosphate concentrations and self-perceived musculoskeletal stiffness and physical limitation.

This retrospective study of 13 adults with XLH supports clinical trial evidence for burosumab as a well-tolerated effective treatment capable of improving serum phosphate concentrations and self-perceived musculoskeletal stiffness when used in a real-world setting. Prior to burosumab, our cohort had suffered numerous musculoskeletal complications and considerable disease burden resulting in impairments in self-perceived stiffness and physical function. All patients experienced early resolution of hypophosphatemia within the first 3 months and significant clinically meaningful improvements in self-perceived stiffness within the first 12 months of burosumab treatment, during which burosumab was well-tolerated. Further long-term prospective real-world studies investigating burosumab use in adults with XLH are needed to further inform clinicians regarding efficacy for clinically relevant endpoints, tolerability, and safety. Since its demonstration in the phase III RCT, we have provided the first real-world evidence indicating burosumab treatment is associated with improved self-perceived stiffness. The underlying mechanisms for this treatment effect require further investigation, including assessment of burosumab effects, if any, on extent of enthesopathy in lower limb load-bearing joints.

## Ethics approval statement

The study protocol and participant consent form were approved by the Northern Sydney Local Health District (NSLHD) Human Research Ethics Committee (HREC) (Ref: 2024/ETH00293).

## Patient consent statement

All participants signed valid informed written consent prior to collection of data for the purpose of this study.

## Data Availability

Datasets were not generated in preparation of this manuscript.
